# A social–ecological analysis of ecosystem services in two different farming systems

**DOI:** 10.1007/s13280-014-0603-y

**Published:** 2015-01-09

**Authors:** Erik Andersson, Björn Nykvist, Rebecka Malinga, Fernando Jaramillo, Regina Lindborg

**Affiliations:** 1 Stockholm Resilience Centre, Stockholm University, 106 91 Stockholm, Sweden; 2 Stockholm Environment Institute, Linnégatan 87D, 115 23 Stockholm, Sweden; 3 Department of Physical Geography and Quaternary Geology, Stockholm University, 106 91 Stockholm, Sweden

**Keywords:** Farming systems, Ecosystem services, Biodiversity, Farmer values, Multifunctional landscapes

## Abstract

In this exploratory study we use existing in situ qualitative and quantitative data on biophysical and social indicators to compare two contrasting Swedish farming systems (low intensity and high intensity) with regard to ecosystem service supply and demand of a broad suite of services. We show that the value (demand) placed on a service is not necessarily connected to the quantity (supply) of the service, most clearly shown for the services recreation, biodiversity, esthetic experience, identity, and cultural heritage. To better capture this complexity we argue for the need to develop portfolios of indicators for different ecosystem services and to further investigate the different aspects of supply and demand. The study indicates that available data are often ill-suited to answer questions about local delivery of services. If ecosystem services are to be included in policy, planning, and management, census data need to be formatted and scaled appropriately.

**Electronic supplementary material:**

The online version of this article (doi:10.1007/s13280-014-0603-y) contains supplementary material, which is available to authorized users.

## Introduction

The ecosystem service concept has gained massive attention from both science and policy as a way to promote sustainable management of ecosystems, natural resources, and landscapes (Daily et al. 2009). However, the lack of knowledge on how to implement and practically use this framework to sustain service benefits is still unexplored with regards to issues like what services should be included in assessments (Reyers et al. 2013), which is the proper scale for management (Scholes et al. 2013), what effects different landscape settings have on service generation (Andersson et al. 2014), and how social and ecological aspects of services can be integrated or disentangled using site-specific data (Reyers et al. 2013). In this study we used two contrasting Swedish farming systems (low intensity and high intensity) to explore how a broad approach to ecosystem service assessment can deepen and structure our understanding of agricultural landscapes. We combined site-specific measures and indicators related to ecosystem service generation with interview material reflecting farmer perceptions and preferences, derived from earlier published research within the Ekoklim program (Stenseke et al. 2012; Nykvist 2014; Andersson and Lindborg 2014; Beilin et al. 2014). The research method was explorative and tested this approach to transdisciplinary assessment by using existing in situ data to examine the different social–ecological dimensions influencing the ecosystem services potentially provided by different landscapes.

Rural landscapes, understood as coupled social–ecological systems, generate different ecosystem services that benefit human well-being and development (Parrott and Meyer 2012). In the sense of ecosystem services, agricultural landscapes can be multifunctional and are increasingly expected to deliver a broad range of services simultaneously (Rabbinge and Bindraban 2012). Ecological and societal feedbacks shape the flow of services and may promote, reduce, or unravel such bundles during the constant negotiation of different trade-offs (Foley et al. 2005; Raudsepp-Hearne et al. 2010; Smith et al. 2012). For example, different drivers of change will affect the composition of services: intensification of farming generally creates landscapes with high output of a few provisioning ecosystem services rather than a broad spectrum of different services (Milestad et al. 2011), while the opposite, abandonment of agricultural landscapes, can lead to loss of traditionally managed pastures and their associated biodiversity (Lindborg et al. 2008; Queiroz et al. 2014). In order to understand and evaluate ecosystem services, and how they interact with certain lifestyles, we need to understand the delivery, beneficiaries, and management of services (Gos and Lavorel 2012). Individuals are likely to hold very different values and combining or generalizing these for integration must be done with extreme caution. The perceived and subjective attractiveness of any landscape will be a combination of the multiple functions it has to offer and the interests of the individual person. This implies that the relationship between the supply of a specific ecosystem service and the demand or appreciation of it is far from straightforward, and context-dependent rather than universal (e.g., Booth et al. 2011).

Our assessment integrated qualitative and spatially explicit quantitative measurements of indicators that can be interpreted in terms of ecosystem service supply and demand, using available data from near-farmhouse and landscape scales. We thus adhere to the description of ecosystem services being defined by the combination of supply of ecological functions, often under the influence of human management, and the demand for these (Costanza et al. 1997). However, while much research focuses on sole indicators for monetary assessments of each service, we discuss how the use of multiple indicators on ecosystem service supply and demand may inform ecosystem services management.

## Study area

**Fig. 1:**
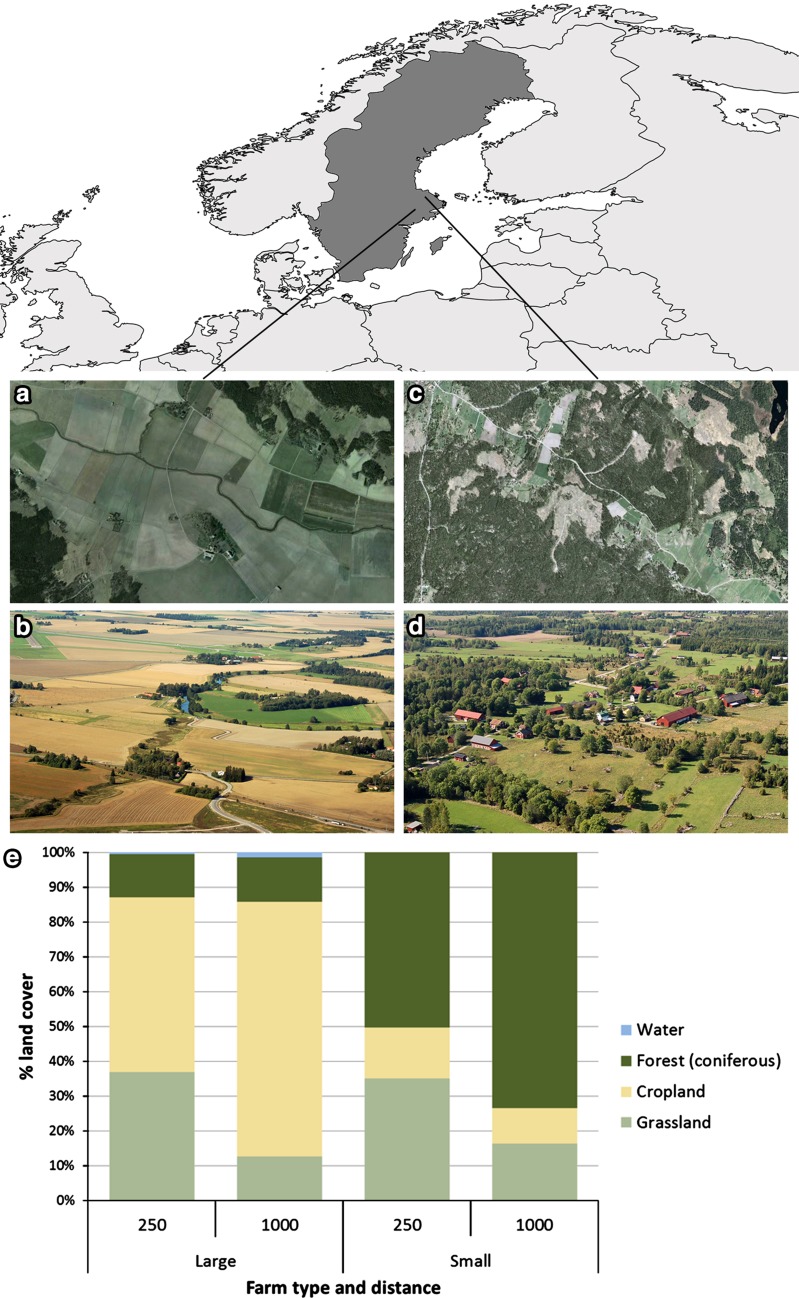
Study area and the two different farming systems. Pictures **a**, **b** show high-intensity farms and **c**, **d** low-intensity farms. **e** Shows the average land cover composition within 250 and 1000 m, respectively, from each farmhouse in the different farming systems

The study area is situated in south-central Sweden in Uppsala County (Fig. 1), an area with fairly homogeneous climate. Despite the high northern latitude, the summers are warm, with July being the warmest month (average maximum temperature of 21 °C), and January the coldest (with an average minimum of −8 °C), with freezing spells that can last a number of consecutive days. Rainfall is higher during the summer months of the year (up to 60 mm/day), while less abundant in winter (up to 25 mm/day), accumulating around 530 mm per year. The two farming systems mainly differ in the proportion crop land (on average 6 % within a 5 km circle around farmhouses in the low-intensity system compared to 44 % around farmhouses in the high-intensity system) and of forest (78–41 % within 5 km from farm houses) surrounding the farms (often, if not always, in part owned and managed by the same farmers). The more forested landscape in north-east is characterized by primarily sandy soils, while soils in the south-west are dominated by clay.

The geophysical conditions differ in the region; high-intensity farms are always located in areas with richer soils and flatter topography, while low-intensity farms are mostly found in remote areas with poorer soils (Strijker 2005; Lindborg et al. 2008).

### The farms

We based our analysis on 16 farms for which we had extensive qualitative data from earlier studies in the Ekoklim program (Stenseke et al. 2012; Beilin et al. 2014; Nykvist 2014). In these studies, eight of the farms were originally randomly drawn from the 100 largest in the intensively managed agricultural area around Uppsala–Enköping–Västerås (approximate center point WGS84 decimal 59.8, 17.5), hereafter “high intensity farms”, and eight were drawn from the 50 smallest farms (hereafter “low intensity farms”) located on the more forested Hållnäs peninsula (approximate center point WGS84 decimal 60.6, 17.9) (Fig. 1). The high-intensity farms had a mean size of 336 ha and the low-intensity farms 13 ha.

## Materials and methods

### Analytical framework: Landscape assessment of ecosystem services

Adopting an approach similar to the UK landscape character assessments (see e.g., Swanwick 2004) we view landscapes as physical manifestations of social–ecological systems, i.e., the results of interacting natural (the influences of geology, soils, climate, flora, and fauna) and cultural (the historical and current impact of land use and management, world views and preferences) factors. A list of ecosystem services can be based on literature reviews, data availability, case-specific needs, issues and trends, local and national policy goals, or knowledge of stakeholders (Malinga et al. 2013). We focused on landscape services, i.e., services that can be used in situ (Lamarque et al. 2011), held to be relevant in the studied landscapes (informed by literature, policy, and previous work with farmers in the two different systems; Nykvist 2014). People’s perceptions and needs turn ecosystem processes and functions into ecosystem services, and these become realized when the end user gets access to the resource. Thus, services were assessed through a set of indicators related to social–ecological factors in earlier studies identified as relevant for the service supply and demand (Table 1, see Electronic Supplementary Material, Tables 10.1007/s13280-013-0603-3 and 10.1007/s13280-013-0603-3 for details and references).

**Table 1:** Indicators connected to ecosystem services generation as they address mediating factors relevant for each service, respectively. Some indicators are used for more than one service, and as the generation of ecosystem services can be influenced by multiple factors most services have more than one indicator. See Electronic Supplementary Material for details

Ecosystem service	Indicator number # (from SI)
1. Pollination	P4, S10
2. Pest control	P4, S3
3. Recreation	P1, P2, S9
4. Biodiversity	P3, P7, P8, S4, S8, S10
5. Food production	P2, P5, S2, S6
6. Timber production	P2, P6, S2, S6
7. Nutrient retention	P10, S3
8. Water availability	P9
9. Esthetic experience	P1, P3, S1, S10
10. Farmer identity	S1, S2, S5, S9, S11
11. Cultural heritage	P11, S4, S7

As our study was exploratory we do not explicitly statistically test or evaluate causal links of the different indicators. Instead, the analysis of ecosystem services was intended to improve conceptual understanding and was guided and constrained by a list of considerations: (1) We wanted a broad set of services representing different groups of ecosystem services defined by TEEB (2010), i.e., regulating, supporting, provisioning, and cultural; (2) Indicators should ideally capture both supply and demand aspects of services, hence we used both biophysical and social indicators to describe different aspects of service generation (cf. de Groot et al. 2010). (3) As we relied on already existing data, i.e., values expressed by beneficiaries (Stenseke et al. 2012; Nykvist 2014; Beilin et al. 2014) and species surveys of birds and vascular plants (Andersson and Lindborg 2014), and publicly available information, we had to choose service indicators for which we could get relevant information; and (4) we wanted spatially explicit information as data that have to be relevant and accessible ecosystem services (cf. Syrbe and Waltz 2012) at either one of two scales: near farmhouse or landscape (within 5 km from farmhouses).

### Landscape specific empirical data

#### Birds and plant surveys

Birds and plants are expected to highlight different aspects of the same landscapes due to differences in scales and environmental drivers they respond to (e.g., Söderström et al. 2001). Both taxa are highly visible parts of any landscape and thus provide an element of biodiversity that people can easily relate to. All farms were surveyed in 2011 (plants) and 2012 (plants and birds) (for details, see Andersson and Lindborg 2014). Bird surveys used the point count method (Bibby et al. 2000) where five survey points were located at and around each farmhouse and surveyed two times: in early May and late May/early June. Vascular plants were surveyed in four habitat types adjacent to all selected farmhouses: forest, semi-natural pasture, grazed ex-arable field, and field margin, with ten randomly selected plots in each habitat.

#### In-depth interviews

Perceptions of the value of ecosystem services were assessed based on earlier conducted three-part, open-ended interviews held with farmers (total field visit 2–3 h). Interviews consisted of both introductory conversation of the history of the farm and farming practices, a recorded semi-structured interview (1–2 h) supported by maps to further facilitate dialog, and additional unrecorded parts where the farmer gave additional in situ information about values and changes in the landscape over time (Nykvist 2014). For our analysis of perceptions of ecosystem services this existing material was coded inductively with open codes classifying patterns related to biodiversity, management, farmers’ relations to nature, values held, and important challenges (Coffey and Atkinson 1996; Patton 2002).

### Publicly available data

Data were extracted from existing GIS-databases (Electronic Supplementary Material Table 10.1007/s13280-013-0603-3) ranging from land cover maps to statistical census information. All secondary data were spatially explicit, but with varying resolutions. Some information was only available at municipal or county level (e.g., average crop and timber production) while other data sets had detailed information (e.g., location and shape of agricultural fields).

### Method for comparing landscapes

**Fig. 2:**
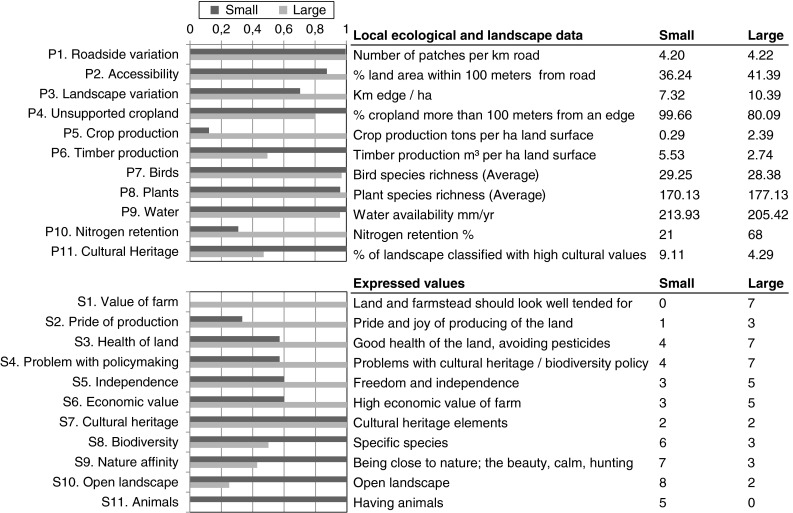
Comparisons between low- and high-intensity farms. The *bars* to the *left* shows normalized differences with the highest value for each variable set to 1. The figures in the two right-most columns show the actual values for each indicator. The *P* variables are either measured at the near-farmhouse scale or at the landscape scale. All *S* variables are measured at the farm level

Both empirical and census data were coded and translated into indicators with values between 0 and 1 (Fig. 2). For the interview data (variables S1–S11, see Electronic Supplementary Material, Table 10.1007/s13280-013-0603-3), emergent patterns on values were further aggregated using selected codes representing core categories of values (sensu Bowen 2008). Each interview was translated to nominal variables stating presence of expressed values in these selected categories. Each indicator was then represented as the number of interviewees expressing the value relative to the total.

For the physical data (variables P1–P11, see Electronic Supplementary Material, Table 10.1007/s13280-013-0603-3), absolute values were normalized to have 1 representing the highest value in this study for each indicator (Fig. 2). In some cases, especially for physical indicators derived from census data, the indicators were constructed based on the combination of several data sources (e.g., average annual increment per municipality (Skogsstyrelsen) x forest area within 5 km from farmhouses (Lantmäteriet) for timber production, see Electronic Supplementary Material Table 10.1007/s13280-013-0603-3). In our approach, indicators could address multiple ecosystem services (Table 1) (Bryan et al. 2011), which created a platform for comparing different understandings and dimensions of the different services. Finally, to further explore the importance of simultaneous analysis of several indicators for each ecosystem service we conducted a literature-based expert assessment of each of the different social and physical indicators (11 each) indicating how these were linked to supply of and demand for a specific service (Electronic Supplementary Material, Tables 10.1007/s13280-013-0603-3 and 10.1007/s13280-013-0603-3).

## Results

### Production landscapes and management

Supply of and demand for several ecosystem services in high-intensity and low-intensity farming systems (Fig. 2) differed, especially on the demand side. The high-intensity farming system generated more crops but still had a sizeable fraction of forest and potential timber production. Nutrient retention, here indicated by the percentage of nitrogen retained in the different sub-catchments, was markedly higher in the high-intensity system (Fig. 2, indicated by P10). Farmers with high-intensity farms felt they constantly had to make trade-off decisions between production of provisioning ecosystem services and the pressure this production put on the environment, especially through nutrient input.

High-intensity farmer (5) “I don’t believe in either or, of either organic or not … I believe that for this to work a middle way is needed. But how to manage that… I mean, you care, this is where you live, and all your neighbors and friends—you don’t want to pollute waters, you do all you can to minimize impacts.”

Both sets of farmers expressed a profound care for the land and the landscape. The owners of the low-intensity farms all had occupations unrelated to farming providing the major part of their income, and being a farmer was valued for the pleasure of being in and interacting with nature (Fig. 2, indicated by S9). In contrast, the owners of the high-intensity farms were professionals with little or no additional income; they valued their independence as farmers, and often viewed regulations on preservation of cultural heritage as chafing (Fig. 2, indicated by S4 and S5). Illustrated by one high-intensity farmer, identity was strongly linked to the pride in producing and selling crops grown on their land.

High-intensity farmer (1): “To be able to sow in the spring, and amble in the field and watch how it grows, that gives me great pleasure […] I then follow the sprouts until they are a decimetre. To see this, you walk there and can see it growing a centimetre or two each day, the strength…”

One of the most explicitly articulated values was the importance of having well-tended farms, where the land itself, together with the buildings and infrastructure, should be in good condition and both look and be economically valuable (Fig. 2, indicated by S1). This is in stark contrast to how the low-intensity farmers value their production landscape (described below).

### Recreation and other non-economic uses of land

The demand for opportunities to hunt and to gather berries and mushrooms was high among low-intensity farmers. They also valued the option to keep small stocks of sheep or beef cows. Animals were often raised for recreational and personal reasons (Fig. 2, indicated by S11) as they provide little economic net income, and the low-intensity farming system had only a handful commercial dairy or beef farmers. This stands in contrast to the high-intensity farms where the few cases of larger stocks of poultry or pigs were complementary parts of professional farming enterprises that specialize in cash crops.

Low-intensity farmer (6)”Nature does work without pesticides. The pesticides have been brought into increase productivity. Perhaps I am not right in this, but if you put plants and animals under stress you lose a lot. A fast growing carrot is not as rich in minerals and vitamins as one that has been allowed to grow slowly. The same for animals, you shouldn’t force a cow to eat too much cereals—they are grass eaters.”

Grazed semi-natural grasslands were more frequent in the low-intensity system (Fig. 2, indicated by P11). All low-intensity farmers engaged in farming for the joy of producing for example high quality meat for the household, and for the clearly stated importance of preserving traditional cultural landscapes. The comparison of road networks in the two systems indicated that the landscape around the high-intensity farms was more easily accessible although the variation in land cover types along the roads was very similar (Fig. 2, indicated by P1–P4).

### Biodiversity

Croplands belonging to the low-intensity farms were almost completely within 100 m from a non-cropland permeable land cover (semi-natural grasslands, forest, fallows, and wetlands), indicating good potential supply of both pollination and natural pest control, earlier demonstrated to be beneficial for agricultural production (Cardinale et al. 2012). In comparison, the high-intensity farms had approximately one-fifth of the total area of cropland more than 100 meters from a non-cropland land cover (Fig. 2, indicated by P4). The landscape surrounding the high-intensity farms was more heterogeneous (Fig. 2, P3) with land cover parcels on average smaller and less contiguous than around the low-intensity farms. In the more forested low-intensity system, farmers often managed the land specifically for the purpose of preserving an open mosaic landscape with high biodiversity.

Low-intensity farmer (5)”We have high priority areas [for biodiversity conservation] here and many plants would disappear if we used artificial fertilizers. The grass would take over and all the little flowers and plants would disappear. […] To me the preservation of the meadows and the flowers is precious”

To the low-intensity farmers conservation meant keeping the forest from expanding, and in some cases actively reclaiming abandoned land. In terms of bird and plant diversity, the farm environment in the low-intensity system had more forest-associated species (on average 11.6 compared to 5.4 species) and compared to the high-intensity farms many bird species associated with agricultural lands were absent despite the presence of fields and active agriculture (on average 4.4–8.5 species) (see Electronic Supplementary Material Tables 10.1007/s13280-013-0603-3, 10.1007/s13280-013-0603-3 for complete species lists). Plants associated with semi-natural pastures were found in comparable numbers in the two systems (mean richness 34.4 species in low and 33.9 in high). The farmers, low intensity more than high intensity, stressed the importance of managing land to maintain high biodiversity, often referring to specific threatened species or groups of species, although few were found in the survey (Fig. 2, S8; Electronic Supplementary Material Tables 10.1007/s13280-013-0603-3, 10.1007/s13280-013-0603-3).

## Discussion

### Two different agricultural landscapes

This study compared two different farming systems by using existing information on both landscape characteristics and farmer perceptions to provide insights about the interplay between supply and demand of ecosystem services in real landscapes. One key finding was that the value (demand) placed on a service is not necessarily or obviously connected to the quantity (supply) of the service, meaning that interpretation of indicators and hence also services per se is complex. This was most clearly shown for the services recreation, biodiversity, esthetic experience, identity, and cultural heritage (Table 2), suggesting that these services can be understood in multiple ways and that different features will attract different people. For example, while the identity of being the care taker of a farm and its surrounding landscape was strong in both systems, it was related to different features and landscape qualities. In contrast, services providing goods with direct market (consensus) value such as timber production or food production showed similar patterns across indicators and much of the service was generally associated with higher value placed on it by the farmers. However, valuation has many dimensions: the greater importance put on these services by the high-intensity farmers is also connected to their identities as professional farmers producing cash crops (Stenseke 2009).

**Table 2:**
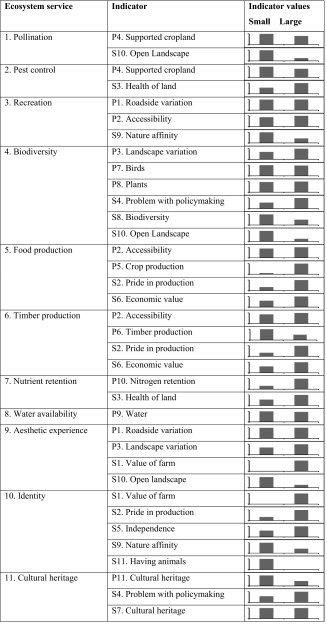
Indicator suites for different ecosystem services and their relative differences in low- and high- intensity farm systems. Differences between systems are site-specific measures, but not statistically tested

In terms of biodiversity (measured as species richness or number of red-listed species), we detected only small differences between high- and low-intensity farms (Fig. 2) and found landscape heterogeneity to be higher in the intensive system. Both these results go against the literature suggesting that more production-oriented landscapes hold less diversity (e.g., Stoate et al. 2009; Tcharntke et al. 2012). Interestingly, farmer perceptions of biodiversity and the value ascribed to it were more in line with the literature; our results showed that more of the farmers on low-intensity farms held biodiversity to be important to them, both at species and landscape levels, while farmers in the intensive system were more worried about negative impacts of management. However, the two systems had very dissimilar species communities, which from a conservation management perspective is important to consider since having both systems within the region helps to increase the overall diversity. Finally, while species communities differed between the two systems we cannot, based on our material, say if this in any way affected the perception of biodiversity.

Results concerning cultural ecosystem services (or aspects of these) such as esthetic experience, cultural heritage, farmer identity, and the appreciation of biodiversity are in general more difficult to interpret (Daniel et al. 2012). The esthetic experience and the value attached to the different landscapes were described and contextualized differently by the two groups of farmers. Low-intensity farmers appreciated aspects of the wider landscape they live in, while high-intensity farmers emphasized the near-farmhouse environment, e.g., buildings, gardens, and infrastructure, which is congruent with earlier studies on farmer perceptions (Stenseke 2009). This could be related to the low-intensity farming system having a higher proportion of semi-natural habitats, highly appreciated both for their biological and cultural values and heritage (Lindborg et al. 2008; Fischer et al. 2008), in the landscape surrounding the farms. In this study, open land was more scarce in the low-intensity farming system and was seen as more precious than in the high-intensity system. One explanation could be that open land is more strongly associated with old traditional management methods and cultural heritage among the low-intensity farmers (cf. Tveit et al. 2006), but it could also be that it is scarce *per se* and provide a welcome variation in the otherwise forested landscape.

### Understanding and connecting supply and demand

Ecosystem services can be a gateway to expand and deepen our understanding of social–ecological systems (Millennium Ecosystem Assessment 2005). To practically use the ecosystem service framework in management, we need to understand the multiple interconnections between physical landscapes and how they are interpreted and used by people (e.g., Cowling et al. 2008). The use of any one, single indicator for a given service will only capture part of the complexity of the social–ecological interplay (Norgaard 2010). Especially when using already existing data, indicators tend to be either biophysical or socioeconomic, and thus be ill-suited to address the social–ecological nature of ecosystem services.

So far, few studies have combined site-specific assessments of supply and demand for ecosystem services (Villa et al. 2014), and few models or frameworks explicitly distinguish changes in the functioning of the ecosystem and human use of such functions (Schulp et al. 2012). Based on our results, we argue that many ecosystem services can be understood only as combinations of biophysical and social indicators. Although we agree with other studies (e.g., Villa et al. 2014) that the social indicators are more related to the demand side of ecosystem services, some social indicators can also be associated with supply and vice versa. When we reviewed our list of indicators, we found demand to be a relevant dimension also for the cases where biophysical indicators could be directly connected to environmental needs, i.e., the indicator addressed a potential environmental problem such as nutrient run-off or crop pests. In the cases where social indicators had a direct influence on supply this was through management and active interaction with the landscape, like animal husbandry, compared to pure demand and perception-related issues like problems with cultural heritage/biodiversity policy or freedom and independence (see discussions in Andersson et al. 2007; Russell et al. 2013). To better understand this co-creation, further research is needed on the selection of supply–demand sets of indicators for services and the scales they are relevant at. Furthermore, there is also a need to evaluate relative strengths and weaknesses of using individual indicators that encompass both supply and demand.

The services we were interested in all had a spatially explicit local relevance, i.e., supply was contingent on accessibility. To capture this aspect, we used both indicators that integrated spatial components (e.g., P4) and accessibility itself through infrastructure (P2), which influences the supply of several of the services, with different implications in different systems. For example, the more extensive infrastructure surrounding the high-intensity farms support professional work (and the realization of services like timber and crop production) rather than being an asset for leisure activities, while in the low-intensity system farmers expressed higher interest in recreational uses and access to outdoor activities such as bird watching, hiking, and hunting (for an in-depth discussion, see Syrbe and Walz 2012). The indicators we used were blunt, and future research could further refine the relevant accessibility dimensions for different services.

### Practical implications

The interpretation of all-encompassing indices is at best tentative. To implement the ecosystem service framework, we need to know which information is needed to answer different questions about ecosystem services, and what different indices actually say. The use of several different indicators for the same service (or the same indicator for multiple services) together can inform more comprehensively on the supply and demand dimensions of each service, and thus in a better way capture complexity and inform local decision making. Through triangulation of different indices research can highlight the often non-linear relations between supply and demand, and how these connections depend on stakeholders. For example, our study shows that the potential for natural pest control is lower in the high-intensity farming system, congruent with other recent studies (e.g., Bommarco et al. 2013). High-intensity farmers also used more pesticides, which could be argued to replace the ecosystem service, but the farmers felt uncomfortable with the high use of pesticides and would prefer to use less. More importantly, analysis of anthropogenic inputs to production systems reveals that maintenance of high levels of production is currently holding many systems in otherwise unstable states, potentially leading to the loss of alternative management options for the future (e.g., Rist et al. 2014). Thus, even though low levels of the natural pest control is currently not a direct problem, a different situation with more of the service and less need for the de facto used pesticides would be preferred, a complexity that could not have been revealed with a single indicator analysis.

To implement the ecosystem service framework in practice, data that capture supply and demand are needed also at local farm scales. Using already existing data and information is often advantageous as it is cost efficient, and standardized regional or national information can enable comparative analysis (Raudsepp-Hearne et al. 2010; Queiroz et al. 2015). However, we show that existing data often are insufficient to capture the complexity of ecosystem service supply and demand, and that information may not be generated at a scale where it can be used to support decision making for farmers or landscape managers. A closer collaboration between research, monitoring, and end users to better capture and interpret information at this scale could also further inform research by providing new data, and support governance of ecosystem services by providing analytical frameworks and tools.

## Electronic supplementary material

Below is the link to the electronic supplementary material.

Supplementary material 1 (PDF 633 kb)
