# Supplementary material for: A social–ecological analysis of ecosystem services in two different farming systems
Source: Ambio. 2015 Jan 9;44(Suppl 1):102–12. doi: 10.1007/s13280-014-0603-y (PMC4288997; doi:10.1007/s13280-014-0603-y)
Supplement: Supplementary file 1 — Supplementary material 1 (PDF 633 kb) [file 13280_2014_603_MOESM1_ESM.pdf]

AMBIO

Electronic Supplementary Material

This supplementary material has not been peer reviewed.

A social-ecological analysis of ecosystem services in two different farming systems

Erik Andersson, Björn Nykvist, Rebecka Malinga, Fernando Jaramillo, Regina Lindborg

Definition of regions around farms for surveys

Aggregated 5 km radius circles around all farmhouses in low and high intensity farms, respectively, constitute the two regions/systems. Some indicators (biodiversity and farmer responses) were assessed at the farm level with eight replicates each of high intensity and low intensity farms.

Nominal variables on farmers values

Fully transcribed interview data from open ended questions of what is valued and important were iteratively coded (Patton 2002, pp. 452–453). Each interview was first coded inductively with open codes, capturing statements on values and challenges associated with emerging themes such as the agricultural landscape, production, lifestyles, livelihoods, nature resources and nature. For each interview coded a growing list of statements of what is valued or of high importance (e.g., challenges) was created. The list of statements from all interviews were then coded a second time to aggregate patterns into broad categories of expressed values, e.g., associated with nature and biodiversity, farming practices, production of crops, or specific species. Each interview was finally coded using selected codes representing core categories of values (Bowen, 2008). In this final step each interview was translated to a nominal variables expressing presence of values in these selected categories (1) or not (0), e.g., "Mentions an open and biodiversity landscape as highly valuable" [yes / no]. Finally, average occurrences of values among these variables were computed (table S4).

Supply and demand of ecosystem services

Basically, biophysical indicators address supply and social indicators demand. However, we found this approach as somewhat incomplete as we found aspects of demand among the biophysical indicators and vice versa. While acknowledging that this is a field that needs further study we distinguished environmental need or mitigation of an environmental problem (e.g. nutrient leakage and eutrophication, table S1) a as a system specific demand, and landscape management or stewardship as a factor contributing to the supply of ecosystem services. The latter is related to the social

indices where an expressed value had a direct physical manifestation in the landscape (e.g. land and farmstead should look well-tended for, often with very specific ideas about what "well-tended" meant, table S2)

References

Bowen, G. A. 2008. Naturalistic inquiry and the saturation concept: a research note. *Qualitative Research* 8: 137–152. doi:10.1177/1468794107085301.

Patton, M. Q. 2002. *Qualitative research & evaluations methods*. 3rd ed. Thousand Oaks, California: Sage Publications.

Table S1 Definitions of Physical ecosystem and landscape variables (P1-P11).

Physical Variable	Short name	Supply, demand	Mapping of ecosystem service and definition of variable
P1. Number of patches per km road	Roadside variation	S	Used as an indicator of landscape variation experienced along roads (within 5 km from farmhouses). The index was calculated as number of patches intersected by or adjacent to all roads and paths, except motorways and railways, divided by total road length. Patches were defined using the land cover classification in the digital vector version of the Property Map (Lantmäteriet 2012). Diversity and heterogeneity are described as positive for landscape appreciation (Dramstad et al., 2006; Folke et al., 2002; Hägerhäll, 1999) and roads and trails are the main conduits for movement across the landscape.
P2. % land surface within 100 meter from road.	Accessibility	S	Used as an indicator of accessibility (see e.g. Groom and Reed, 2001; Syrbe and Walz, 2012) and calculated based on all roads and paths within 5 km from farmhouses, except motorways and railways. Road information from the digital vector version of the Property Map (Lantmäteriet 2012).
P3. Km edge per ha.	Landscape variation	S	Used as an indicator of landscape structural composition. Calculated as total length of edges per ha land surface (within 5 km from farmhouses). Edges were defined using the land cover classification in the digital vector version of the Property Map (Lantmäteriet 2012). The indicator addresses perceived diversity (with positive connotations for cultural values, see e.g. Syrbe and Walz, 2012) as well as a rough estimate of habitat support functions (landscape diversity vs.

			fragmentation).
P4. % cropland less than 100 meters from a non-cropland edge other than water or impervious surfaces.	Supported cropland	S, D	Used as an indicator both for pollination and pest control potential as longer distances to semi-natural habitats could have negative consequences for pollination and pest control. Calculated for all cropland within 5 km from farmhouses. Cropland was identified from the 2012 iteration of Swedish IACS (Integrated Agricultural Control System Databases, provided by the Swedish Board of Agriculture) and edges were defined using the land cover classification in the digital vector version of the Property Map (Lantmäteriet 2012). Pollination and pest control have been demonstrated to be spatially contingent (Blitzer et al. 2012) and 100 meters is a very conservative proxy for pollinator mobility (Steffan-Dewenter et al. 2002; Bailey et al. 2014).
P5. Crop production tons / ha land surface and year.	Crop production	S	Used as an indicator of average crop production potential of the landscape if not farm and proportionate to the amount of cropland (within 5 km from farmhouses). We chose the most common crop, autumn wheat, as proxy. Calculations are based on the County specific average yearly yield per ha cropland between 2006 and 2011 (SCB, http://www.statistikdatabasen.scb.se/pxweb/sv/ssd/START__JO__JO0601/SkordarL/?rxid=65e8fb76-d8cb-44eb-a046-5bf27eb2196f downloaded August 2013) and cropland areas (Swedish IACS 2012), divided by total land area.
P6. Timber production m3 per ha land	Timber production	S	Used as an indicator of timber production potential and proportionate to the amount of

surface and year.			forest within 5 km from farmhouses. Calculations are based on the County specific average yearly increment per ha forest (across species and age classes) between 2006 and 2010 (Skogsstyrelsen, http://www.skogsstyrelsen.se/en/AUTHORITY/Statistics/Statistical-Yearbook-/ downloaded August 2013) and forest area (Property map, Lantmäteriet 2012), divided by total land area.
P7. Bird species richness.	Birds	S	Used as an indicator of biodiversity. The average is calculated based on 16 farm-specific surveys (see Andersson and Lindborg, 2014 for more information) and sampled within 300 meters from farmhouses. Bird diversity is both a direct experiential service (e.g. Hedblom et al. 2014) and the foundation for many other ecosystem services (e.g. Wenny et al. 2011).
P8. Vascular plant species richness.	Plants	S	Used as an indicator of biodiversity. The average is calculated based on 16 farm-specific surveys (see Andersson and Lindborg, 2014 for more information) done within 1 km from farmhouses. Plants carry many meanings; some flowering plants are strongly associated semi-natural grasslands and cultural heritage (Quétier et al. 2007).
P9. Water availability	Water	S, D	Used as an indicator of water turn over and availability in the landscapes. Availability was measured as mean annual flow (m^3/s) per ha land surface, based on model values for each sub-catchment (SMHI, http://vattenwebb.smhi.se/modelarea/ downloaded November 2012) and then averaged for the two systems. The model is built and trained on a data set for the years

			1990 to 2010.
P10. Nitrogen retention in %	Nutrient retention	S, D	Used as an indicator of the capacity of the landscape and water bodies to capture and retain nutrient input between the emission point in the agricultural plot and the discharge of the water course in the Baltic Sea (a combination of plant uptake, leaching, denitrification in the soil and in lakes and streams, primary production and mineralisation). This retention was calculated as: Retention (%) = $(N_b-N_n)/N_b$ where N_b is the agricultural Nitrogen load due to agriculture in the corresponding basin and N_n is the final corresponding nutrient load being discharged into the Baltic Sea which is left after retention in the landscape as modeled by SMHI (http://vattenwebb.smhi.se/modelarea/ downloaded November 2012). Calculated per sub-catchment and averaged for the two systems.
P11. % of open land classified as semi-natural grassland	Cultural heritage	S	Used as an indicator of traditional land-uses such as hay making and cattle grazing. Semi-natural grasslands within 5 km from farmhouses were identified from the Swedish grassland survey (TUVA 2004, http://www.jordbruksverket.se/etjanster/etjanster/tuva/laddanerkartskikt.4.2ce1c8ad1213e6b28d48000866.html downloaded August 2013) and open land (including crop fields) were defined using the land cover classification in the digital vector version of the Property Map (Lantmäteriet 2012). In Sweden semi-natural grasslands are strongly associated with cultural landscapes, especially

			in agricultural systems (Lindborg et al. 2008; Emanuelsson 2009). The indicator captures both supply of experiential services and opportunities to self-fulfillment in terms of upholding traditions (Stenseke 2009).

Table S2 Definitions of social values variables (S1-S11).

Social Variable	Short name	Supply, demand	Valuation process and definition of variable (Description of pattern in data)
S1. Land and farmstead should look well-tended for	Value of farm	S, D	Farmstead should look tidy and well-tended as this expresses the economic value and health of the land and the farmstead. The expression of the farm as valuable in terms of its visual and aesthetic beauty is a pattern commonly found in studies of farmers (Boonstra et al, 2011).
S2. Pride and joy of producing from the land	Pride in production	D	Expressed pride and joy over being farmer that produce of the land. Identity of being a farmer that live of the land, and produce a product of value for society. This pattern has previously been described by, e.g. Ahnström (2009) and illustrates how farmers value generating demand for production ecosystem services.
S3. Good health of the land, avoiding pesticides	Health of land	S, D	Expresses clearly a value and care for the health of the land. Importance of not destroying it, leaving a healthy land to next generation. E.g., that one wants to avoid pesticides if possible. Key theme in how farms manage trade-offs between different values described also by Nykvist (2014).
S4. Problems with cultural heritage / biodiversity policy	Problem with policymaking	D	Express the important challenges or even negative values towards regulations. E.g. cultural heritage items and regulation that inhibit farming, or biodiversity protection as infringing on farming practices. The pattern is strongly linked to the perceived value of being independent, with aversion to top down management and regulation, but in this case expressed explicitly as an eversion to focus on generating cultural heritage and biodiversity values in the landscape (See further, Nykvist, 2014).
S5. Freedom and independence	Independence	D	Expressed value of freedom of being a farmer. The independence of being one's own boss. The freedom and deep satisfaction that farming means in terms of the type of occupation. Key theme in farmer identity described by Nykvist (2014).

S6. High economic value of farm	Economic value	S, D	Expressed motivation being achieving a high economic value of farm (land and farmhouse) per se. That the high value of the farm is a goal and something valuable in itself. Closely related to another strong pattern of the economic profitability of farming described by Stenseke (2006) and many others.
S7. Cultural heritage elements valued	Cultural heritage	S, D	Expresses cultural heritage items that potentially enhances ecosystem functioning, such as stone walls, hedge rows, semi-natural pastures, old barns etc. as valuable and worth preserving, and that this is an important aspect of managing the agricultural landscape. Pattern is similar to the pattern identified by Stenseke (2006) on values assigned to preserving semi-natural grasslands.
S8. Specific species and groups of species valued	Biodiversity	D	Mentions and values specific species (or groups of species) because of their intrinsic value, their interest in biodiversity or nature. Pattern clearly and explicitly describing value of biodiversity.
S9. Being close to nature; the beauty, calm, hunting	Nature affinity	D	Valuing being close to nature, the beauty and calm, the experiences you get. Enjoying activities such as hunting, mushroom or berry picking. The lifestyle of living in rural landscape with its natural resources. Pattern is a complex appreciation of nature and care for the land described also by Boonstra et al. (2011).
S10. Open landscape	Open landscape	S, D	Open landscape is valued as a landscape features. Including explicit mentioned values of an open traditional agricultural landscape being linked to biodiversity, and therefore valuable. The pattern to value the open landscape is also linked to preserving particular cultural heritage elements (Stenseke 2006)
S11. Having animals	Animals	S, D	Having animals is expressed as value in itself. That one enjoys the practices and work associated with having animals, and also including the value of having them in order to have a varied agricultural landscape with animals. Pattern clearly and explicitly

			describing value of having animals.

References for Tables S1 and S2

Ahnström, J. 2009. Farmland Biodiversity - in the Hands and Minds of the Farmers. Doctoral Thesis. Swedish University of Agricultural Sciences.

Andersson, E. and R. Lindborg. 2014. Different agricultural landscapes and the conservation of birds and plants. *PLoS one, accepted pending revisions*

Bailey, S., F. Requier, B. Nusillard, S. P. M. Roberts, S. G. Potts and C. Bouget. 2014. Distance from forest edge affects bee pollinators in oilseed rape fields. *Ecology and Evolution*: n/a–n/a. doi:10.1002/ece3.924.

Blitzer, E. J., C. F. Dormann, A. Holzschuh, A-M. Kleind, T. A. Rand and T Tscharntke. 2012. Spillover of functionally important organisms between managed and natural habitats. *Agriculture, Ecosystems & Environment* 146: 43–34. doi:10.1016/j.agee.2011.09.005.

Boonstra, W. J., J. Ahnström and L. Hallgren. 2011. Swedish Farmers Talking about Nature - A Study of the Interrelations between Farmers' Values and the Sociocultural Notion of Naturintresse. *Sociologia Ruralis* 51: 420–435. doi:10.1111/j.1467-9523.2011.00547.x.

Dramstad, W. E., M. S. Tveit, W. J Fjellstad and G. L. A. Fry. 2006. Relationships between visual landscape preferences and map-based indicators of landscape structure. *Landscape and Urban Planning* 78: 465–474. doi:DOI: 10.1016/j.landurbplan.2005.12.006.

Emanuelsson, U. 2009. *The rural landscapes of Europe – How man has shaped European nature*. Stockholm: Forskningsrådet Formas.

Folke, C., S. Carpender, T. Elmqvist, L. Gunderson, C.S. Holling, B. Walker, J. Bengtsson, F. Berkes, et al. 2002. *Resilience and sustainable developement: building adaptive capacity in a world of transformations*. Stockholm: Swedish Environmental Advisory Council.

Groom, G. and T. Reed, ed. 2001. *Strategic landscape monitoring for the Nordic countries*. Copenhagen: Nordic Council of Ministers.

Hedblom, M., E. Heyman, H. Antonsson and B. Gunnarsson. 2014. Bird song diversity influences young people's appreciation of urban landscapes. *Urban Forestry & Urban Greening*. doi:10.1016/j.ufug.2014.04.002.

Hägerhäll, C. 1999. The Experience of Pastoral Landscapes. Swedish University of Agricultural Sciences.

Lantmäteriet. 2012. Fastighetskartan. Lantmäteriet.

Lindborg, R., J. Bengtsson, Å. Berg, S. A. O. Cousins, O. Eriksson, T. Gustafsson, K. P. Hasund, L. Lenoir et al. 2008. A landscape perspective on conservation of semi-natural grasslands. *Agriculture, Ecosystems and Environment* 125: 213–222. doi:10.1016/j.agee.2008.01.006.

Quétier, F., S. Lavorel, W. Thuiller and I. Davies. 2007. Plant-trait-based modeling assessment of ecosystem-service sensitivity to land-use change. *Ecological Applications* 17. Ecological Society of America: 2377–2386.

Steffan-Dewenter, I., U. Munzenberg, C. Burger, C. Thies and T. Tscharntke. 2002. Scale-dependent effects of landscape context on three pollinator guilds. *Ecology* 83: 1421–1432.

Stenseke, M. 2006. Biodiversity and the local context. Linking seminatural grassland and their future use to social aspects. *Environmental Science & Policy* 9: 350–359. doi:10.1016/j.envsci.2006.01.007.

Stenseke, M. 2009. Local participation in cultural landscape maintenance: Lessons from Sweden. *Land Use Policy* 26: 214–223. doi:10.1016/j.landusepol.2008.01.005.

Syrbe, R-U. and U. Walz. 2012. Spatial indicators for the assessment of ecosystem services: Providing, benefiting and connecting areas and landscape metrics. *Ecological Indicators* 21: 80–88. doi:10.1016/j.ecolind.2012.02.013.

Wenny, D. G., T. L. DeVault, M. D. Johnson, D. Kelly, C. H. Sekercioglu, D. F. Tomback, and C. J. Whelan. 2011. The Need to Quantify Ecosystem Services Provided by Birds. *The Auk* 128. University of California Press: 1–14. doi:10.1525/auk.2011.10248.

Table S3 Species list for bird species, including red list status and habitat preferences.

Bird species	Low intensity farms	High intensity farms	Red list	Habitat
Allauda arvensis	x	x	x	Open
Anas crecca	x			Water
Anas platyrhynchos	x	x		Water
Anser anser		x		Open
Anthus trivialis	x	x		Forest
Apus apus	x	x		Open
Buteo buteo	x	x		Open
Carduelis cannabina		x	x	Open
Carduelis carduelis	x	x		Open
Carduelis chloris	x	x		Mixed
Carduelis spinus	x	x		Forest
Certhia familiaris	x	x		Forest/Woodland
Columba oenas		x		Forest
Columba palumbus	x	x		Forest
Corvus corax	x	x		Forest
Corvus corone	x	x		Mixed
Corvus monedula	x	x		Open
Cuculus canorus	x	x		Forest/Woodland
Cygnus Cygnus		x		Water/wetlands
Dendrocopus major	x	x		Trees
Dendrocopus minor		x		Forest
Dryocopus martius	x			Forest
Emberiza citronella	x	x		Open
Emberiza schoeniclus		x		Water/wetland
Erithacus rubecula	x	x		Forest
Ficedula hypoleuca	x	x		Forest
Fringilla coelebs	x	x		Mixed
Gallinago gallinago	x	x		Open
Grus grus	x	x		Forest
Hippolais icterina	x	x		Forest
Hirundo rustica	x	x		Open
Jynx torquilla	x		x	Mixed
Lanius collurioides		x		Open

Loxia curvirostra	x			Forest
Luscinia luscinia		x		Forest
Motacilla alba	x	x		Open
Numenius arquata		x	x	Open
Oenanthe oenanthe	x	x		Open
Pandion haliaeetus		x		Forest/water
Parus ater	x	x		Forest
Parus caeruleus	x	x		Mixed
Parus crestatus	x			Forest
Parus major	x	x		Mixed
Passer domesticus & montanus		x		Open
Phasianus colchicus		x		Open
Phoenicurus phoenicurus		x		Forest
Phylloscopus collybita	x			Forest/Woodland
Phylloscopus sibilatrix	x			Forest
Phylloscopus trochilus	x	x		Forest
Phylloscopus trochilus	x	x		Forest
Pica pica	x	x		Mixed
Picus viridis	x	x		Mixed
Pluvialis apricaria		x		Open
Prunella modularis	x			Forest
Pyrrhula pyrrhula	x			Forest
Regulus regulus	x	x		Forest
Sitta europaea	x	x		Trees
Sturnus vulgaris	x	x		Open
Sylvia atricapilla	x	x		Forest
Sylvia communis	x	x		Open
Sylvia curruca	x	x		Open
Tetrao tetrix	x			Forest
Tringa ochropus	x			Forest
Troglodytes troglodytes	x	x		Forest
Turdus iliacus	x	x		Mixed
Turdus merula	x	x		Forest/Woodland
Turdus philomenos	x	x		Forest
Turdus pilaris	x	x		Mixed
Turdus viscivorus	x			Forest
Vanellus vanellus		x		Open

Table S4 Species list for vascular plant species, including red list status and whether or not the species is associated with either ruderal lands or traditional management practices.

**Plant species**	**Low intensity farms**	**High intensity farms**	**Red list**	**Ruderal species**	**Grassland species**
Abies alba	x	x			
Acer platanoides	x	x			
Achillea millefolium	x	x			x
Achillea ptarmica		x			
Actaea spicata	x	x			
Aegopodium podagraria		x			
Agrimonia eupatoria	x	x			x
Agrostis capillaris		x			x
Agrostis gigantea		x			
Agrostis stolonifera	x	x			
Ajuga pyramidalis	x				x
Alchemilla glaucescens	x				x
Alisma plantago-aquatica	x	x			
Allium oleraceum	x	x			x
Allium x hollandicum	x				
Alnus glutinosa					
Alopecurus aequalis	x	x			
Alopecurus geniculatus	x	x			
Alopercurus pratensis	x	x			
Anchusa arvensis	x	x			
Anemone nemorosa	x	x			
Angelica sylvestris	x	x			
Antennaria dioica					x
Anthemis tinctoria	x	x			
Anthoxanthum odoratum	x	x			x
Anthriscus sylvestris		x		x	
Anthyllis vulneraria	x				x
Aquilegia vulgaris	x	x			
Arabis glabra	x	x			
Arctium minor		x			
Arctium tomentosum		x			
Arenaria serpyllifolia		x			x
Armoracia rusticana		x			
Arrhenatherum elatius		x		x	
Artemisia vulgaris	x	x			x
Athyrium filix-femina		x			
Avena sativa		x			
Barbarea vulgaris	x	x			
Berberis vulgaris	x	x			

Betula pendula	x	x			
Betula pubescens	x				
Bidens tripartita	x	x		x	
Bistorta vivipara	x				x
Botrychium lunaria	x		x		x
Brachypodium pinnatum	x				
Brachypodium sylvaticum	x				
Briza media		x			x
Bromopsis inermis					x
Bromus hordeaceus					x
Bunias orientalis	x	x			
Calamagrostis arundinacea		x		x	
Calamagrostis canescens	x	x			
Calluna vulgaris	x	x			
Caltha palustris	x				
Calystegia sepium				x	
Camomilla suaveolens	x	x			
Campanula glomerata	x	x			
Campanula latifolia	x	x			
Campanula patula	x				
Campanula persicifolia		x			
Campanula rapunculoides	x	x			
Campanula rotundifolia	x	x			x
Capsella bursa-pastoris	x	x		x	
Cardamine pratensis	x				x
Carduus crispus		x			
Carex acuta		x			
Carex canescens	x	x			
Carex digitata	x	x			
Carex disticha	x				
Carex echinata	x	x			x
Carex elata	x				
Carex elongata	x				
Carex flacca	x	x			
Carex flava	x				
Carex hartmanii	x		x		
Carex hirta	x	x			
Carex hostiana	x		x		
Carex nigra	x	x			
Carex ovalis	x	x			
Carex pallescens	x	x			x
Carex panicea	x	x			
Carex pilulifera	x	x	x		x
Carex rostrata		x			

Carex spicata	x	x			x
Carex vaginata					
Carex vulpina					
Carlina vulgaris	x	x			
Carum carvi		x		x	
Centaurea cyanus	x	x			
Centaurea jacea	x	x			x
Centaurea scabiosa	x	x			
Cerastium fontanum	x	x			x
Chelidonium majus	x	x		x	
Chenopodium album		x			
Chenopodium rubrum		x			
Chenopodium suecicum	x	x			
Cichorium intybus	x				
Cirsium arvense	x	x		x	
Cirsium helenioides	x				
Cirsium palustre	x	x			
Cirsium vulgare		x		x	
Consolida regalis	x	x	x		
Convallaria majalis		x			
Convolvulus arvense	x	x		x	
Corylus avellana	x	x			
Cotoneaster integerrimus		x			x
Crataegus monogyna	x	x			
Crepis paludosa	x				
Crepis praemorsa					x
Crepis tectorum	x	x			
Cystopteris fragilis	x	x			
Dactylis glomerata	x	x			
Dactylorhiza maculata	x				
Dactylorhiza virides	x				
Danthonia decumbens	x				x
Daphne mezereum	x				
Deschampsia cespitosa	x	x			
Deschampsia flexuosa	x	x			
Dianthus deltoides	x	x			x
Dryopteris carthusia	x	x			
Dryopteris filix-mas		x		x	
Elymus caninus	x	x		x	
Elytrigia repens	x	x			
Epilobium angustifolium		x		x	
Epilobium ciliatum	x	x			
Epilobium montanum	x	x			
Epipactis helleborine	x				
Equisetum arvense		x			

Equisetum fluviatile	x	x			
Equisetum heymale	x				
Equisetum palustre					
Equisetum pratensis	x			x	
Equisetum sylvaticum	x				
Eriophorum angustifolium	x				
Eriophorum gracile	x				
Eriophorum vaginatum					
Erodium cicutarium	x				
Erysimum cheiranthoides		x			
Euphorbia helioscopia		x			
Euphrasia stricta		x	x		x
Fagus sylvatica	x	x			
Fallopia convolvulus		x			
Fallopia dumetorum	x	x			
Festuca arundinacea	x				
Festuca ovina	x	x			x
Festuca pratensis		x			
Festuca rubra	x	x			x
Filipendula ulmaria	x	x		x	
Filipendula vulgaris	x	x			x
Fragaria vesca	x	x			x
Frangula alnus	x	x			
Fraxinus excelsior	x	x			
Fumaria officinalis	x	x			
Galeopsis bidifa					
Galeopsis ladanum		x	x		
Galeopsis speciosa		x			
Galeopsis tetrahit	x	x			
Galium album		x			x
Galium aparine	x	x			
Galium boreale	x	x			
Galium palustre		x			
Galium spurium	x	x			
Galium uliginosum	x	x			x
Galium verum		x			x
Gentianella campestris	x		x		x
Gentianella campestris			x		x
Geranium pratense		x			
Geranium pusillum	x	x		x	
Geranium robertianium	x	x			
Geranium sanguineum	x				
Geranium sylvaticum	x	x		x	
Geum rivale	x	x			

Geum urbanum	x	x			
Glechoma hederacea	x	x			
Glyceria fluitans	x	x			
Glyceria maxima					
Gnaphalium sylvaticum	x				x
Gnaphalium uliginosum	x	x			
Gymnadenia conopsea	x				
Gymnocarpium dryopteris	x	x			
Helianthemun nummularium	x	x			x
Helictotrichon pratense	x	x			
Helictotrichon pubescens	x	x			
Hepatica nobilis	x	x			
Heracleum sphondylium		x			
Hesperis matronalis	x	x			
Hieracium sect. Hieracium	x	x			
Hieracium sect. Vulgata	x	x			x
Hieracium umbellatum	x	x			
Hierochloë odorata	x				
Hippophaë rhamnoides	x				
Hypericum maculatum		x			
Hypericum perforatum	x	x			x
Hypochoeris maculata					
Inula salicina	x	x			x
Iris pseudacorus					
Juncus alpinoarticulatus	x				
Juncus articulatus	x				
Juncus bufonius	x				
Juncus compressus	x				
Juncus conglomeratus		x			
Juncus effusus	x	x			
Juncus filiformis	x				
Juniperus communis	x	x			
Knautia arvensis		x			
Lactuca serriola	x	x			
Lamium hybridum		x			
Lamium hybridum		x			
Lamium purpureum		x			
Lamium sp.	x	x			
Lapsana communis	x	x			
Laserpitium latifolium					
Lathyrus linifolius	x	x			
Lathyrus pratensis	x	x			x
Lathyrus vernus	x				x
Leontodon autumnalis	x	x			x

Leucanthemum vulgare	x	x			x
Linnaea borealis	x				x
Linum catharticum	x				
Listera ovata					x
Lolium perenne		x			
Lonicera periclymenum	x	x		x	
Lonicera xylosteum	x	x			
Lotus corniculatus		x			
Luzula campestris	x	x			x
Luzula multiflora	x	x			x
Luzula pallescens	x				
Luzula pilosa		x			
Lychnis flos-cuculi	x				
Lychnis viscaria	x				
Lycopodium clavatum	x				x
Lysimachia thyrsiflora	x				
Lysimachia vulgaris	x				
Maianthemum bifolium	x	x		x	
Malus domestica	x	x			
Malus sylvestris					
Malva moschata	x	x			
Matricaria matricarioides	x				
Medicago lupulina		x			
Medicago sativa		x			x
Medicago sativa	x	x			
Melampyrum nemorosum	x				
Melampyrum pratense	x				
Melampyrum sylvaticum	x	x			
Melica nutans	x	x			
Mentha arvensis		x			
Mentha x verticillata	x				
Milium effusum		x			
Moehringia trinervia	x	x			
Molinia caerulea					
Monotropa hypopitys	x				
Mycelis murialis	x	x			
Myosotis arvensis	x	x			
Myosotis laxa		x		x	
Myosotis ramossisima		x			
Myosotis sylvatica	x	x			x
Myrica gale	x				
Nardus stricta	x				
Neottia nidus-avis					x
Origanum vulgare	x	x			
Oxalis acetosella		x			

Papaver dubium	x	x			
Paris quadrifolia		x			
Persicaria hydropiper	x				
Persicaria lapathifolia		x			
Persicaria maculosa		x			
Phagmites australis	x				x
Phalaris arundinacea	x			x	
Phleum phleoides		x			
Phleum pratense	x	x			
Phleum pratense	x				
Picea abies	x	x			
Pilosella lactucella	x	x			x
Pilosella officinarum	x	x			
Pimpinella saxifraga	x	x			x
Pinus sylvestris	x	x			
Plantago lanceolata	x	x			x
Plantago major		x		x	
Plantago media	x	x			x
Platanthera bifolia	x				x
Platanthera chlorantha	x	x			
Poa annua		x		x	
Poa compressa	x	x			
Poa nemoralis	x	x			
Poa pratensis		x		x	
Poa trivialis	x	x			
Polygala vulgaris	x	x		x	
Polygonatum odoratum	x	x			
Polygonum aviculare	x	x		x	
Polypodium vulgare	x	x			
Populus tremula	x	x			
Potentilla anserina	x	x		x	
Potentilla argentea	x	x			x
Potentilla erecta	x	x			
Potentilla palustris	x				
Potentilla reptans		x			x
Primula farinosa	x		x		x
Primula veris	x	x			x
Prunella vulgaris	x	x			
Prunus avium	x	x			
Prunus domestica	x				
Prunus padus	x	x			
Prunus spinosa	x	x			
Pteridium aquilinum		x			
Pyrola chloranta	x	x			
Pyrola rotundifolia	x				

Quercus robur	x	x			
Ranunculus acris	x	x			
Ranunculus auricomus	x	x			x
Ranunculus bulbosus	x				x
Ranunculus flammula	x	x			
Ranunculus polyanthemos	x	x			x
Ranunculus repens		x			
Ranunculus sceleratus		x			
Rhamnus cathartica	x	x			
Rhinanthus minor	x	x			x
Ribes alpinum	x				
Ribes nigrum	x				
Ribes rubrum	x				
Ribes spicatum					
Ribes uva-crispa		x			
Rosa canina	x	x			
Rosa dumalis	x	x			
Rosa sherardi	x	x			
Rosa villosa	x	x			
Rubus idaeus	x	x			
Rubus saxatilis	x	x			
Rumex acetosa	x	x			x
Rumex acetosella	x	x			x
Rumex crispus	x	x			
Rumex longifolius		x			
Rumex obtusifolius	x			x	
Sagina procumbens	x				
Salix caprea	x	x			
Salix cinerea	x	x			
Salix myrsinifolia s	x				
Salix pentandra	x				
Salix repens		x			
Sambucus racemosa	x	x			
Sanicula europaea					
Satureja acinos	x	x			x
Satureja vulgaris	x				
Saxifraga granulata					x
Scleranthus annuus		x			x
Scleranthus perennis		x			
Scorzonera humilis	x	x			x
Scrophularia nodosa	x	x			
Sedum acre		x			x
Sedum sexangulare	x	x			
Sedum telephium		x			
Selinum carvifolia	x				

Senecio sylvaticus		x			
Senecio viscosus		x		x	
Senecio vulgare	x	x		x	
Serratula tinctoria	x		x		x
Seseli libanotis		x			
Sesleria caerulea					
Silene dioca					
Silene nutans		x			x
Sinapsis arvensis		x			
Solanum dulcamara	x	x			
Solidago virgaurea	x	x			
Sonchus arvensis	x	x			
Sonchus asper		x			
Sonchus oleraceus		x			
Sorbus aucuparia	x	x			
Sorbus intermedia	x	x			
Spiraea japonica					
Spiraea sp.	x	x			
Stachys byzantina	x				
Stachys macrantha		x			
Stachys palustris		x			
Stachys sylvatica	x	x		x	
Stellaria graminea	x	x			x
Stellaria media		x			
Stellaria nemorum		x			
Stellaria palustris	x				
Succisa pratensis	x	x			x
Symphytum asperum					
Symphytum uplandicum		x			
Syringa vulgaris		x			
Tanacetum macrophyllum	x				
Tanacetum vulgare	x	x		x	
Thalictrum flavum	x				
Thalictrum simplex	x		x		x
Thlaspi arvense		x			
Thlaspi caerulescens		x			
Thymus serpyllum	x	x			x
Tilia cordata					
Torilis japonica	x	x			
Tragopogon pratensis	x	x			x
Trientalis europaea					
Trifolium arvense		x			x
Trifolium aureum		x			x
Trifolium hybridum	x	x			

Trifolium medium	x				
Trifolium montanum	x	x	x		x
Trifolium pratense	x	x			x
Trifolium repens	x	x		x	
Triglochin palustre	x				
Tripleurospermum perforatum		x			
Triticum aestivum	x	x			
Tussilago farfara	x	x		x	
Ulmus glabra	x	x	x		
Urtica dioica	x	x		x	
Vaccinium myrtillus		x			
Vaccinium uliginosum	x	x			
Vaccinium vitis-idaea	x	x			
Valeriana sambucifolia		x			
Verbascum nigrum		x			
Verbascum thapsus	x	x			
Veronica agrestis		x			
Veronica arvensis	x	x			
Veronica beccabunga	x				
Veronica chamaedrys	x	x			x
Veronica officinalis		x			x
Veronica persica		x			
Veronica scutellata	x				
Veronica serpyllifolia		x			
Veronica spicata	x	x			
Viburnum opulus	x	x			
Vicia cracca		x			
Vicia hirsuta	x	x			
Vicia sepium	x	x			
Vicia sylvatica		x			
Vicia tetrasperma		x			x
Vincetoxicum hirundinaria	x	x			
Viola arvensis	x	x			
Viola canina		x			x
Viola hirta		x			
Viola mirabilis		x			
Viola palustris	x	x			
Viola riviniana	x	x			
Viola tricolor		x			x
